# Minor allele of *GJA1* gene polymorphism is associated with higher heart rate during atrial fibrillation

**DOI:** 10.1038/s41598-021-82117-3

**Published:** 2021-01-28

**Authors:** Sho Okamura, Yuko Onohara, Hidenori Ochi, Takehito Tokuyama, Naoya Hironobe, Yosaku Okubo, Yoshihiro Ikeuchi, Shunsuke Miyauchi, Kazuaki Chayama, Yasuki Kihara, Yukiko Nakano

**Affiliations:** 1 Division of Frontier Medical Science, Department of Cardiovascular Medicine, Programs for Biomedical Research, Graduate School of Biomedical Science, Hiroshima University, 1-2-3 Kasumi, Minami-ku, Hiroshima, 734-8551 Japan; 2 Department of Health Management, Hiroshima Red Cross Hospital & Atomic-Bomb Survivors Hospital, Hiroshima, Japan; 3 Department of Gastroenterology and Metabolism, Biomedical Sciences, Graduate School of Biomedical and Health Sciences, Hiroshima University, Higashihiroshima, Japan

**Keywords:** Cardiology, Medical research

## Abstract

Atrial fibrillation (AF) tachycardia causes heart failure and requires more attention. The genetic background of individual heart rate (HR) variations during AF are unclear. We hypothesized that HR-associated single nucleotide polymorphisms (SNPs) reported in Genome-Wide Association Studies (GWAS) are also associated with HR during AF. We enrolled patients with persistent AF (311 for screening and 146 for replication) who underwent AF ablation and were genotyped for the 21 h-associated SNPs reported in GWAS. The patients underwent 24-h Holter monitoring before AF ablation and electrophysiological study after AF ablation during sinus rhythm. Only the *GJA1* SNP rs1015451 (T>C) was significantly associated with total HR (TT 110,643 ± 17,542 beats/day, TC 116,350 ± 19,060 beats/day, CC 122,163 ± 25,684 beats/day, *P* = 8.5 × 10^−4^). We also confirmed this significant association in the replication set. The intra-atrial conduction was faster in AF patients with the *GJA1* minor allele than in those without it. Multivariate analysis revealed the presence of a *GJA1* SNP rs1015451 additive model, female gender, lower left ventricular ejection fraction, and higher 1:1 atrioventricular nodal conduction were independently associated with higher HR during AF. The *GJA1* SNP might be a new genetic marker for AF tachycardia.

## Introduction

Atrial fibrillation (AF) is the most common cardiac arrhythmia that causes tachycardia^1^. There are several treatment strategies for AF. Rate control is one of the most important baseline AF therapies regardless of the stage of AF, and it is used to either prevent the development of heart failure or reduce the symptoms^2,3^. The Swedish Heart Failure Registry reported that in AF heart failure patients with a reduced ejection fraction, a heart rate (HR) > 100 beats/min was associated with a higher mortality, and β-blocker use was associated with a reduced mortality^4^.

AF is also the most common cause of tachycardia-induced cardiomyopathy in patients without a history of structural heart disease^5^. Adequate rate control can reduce the risk of tachycardia-induced cardiomyopathy and worsening heart failure^6^. However, the HR varies during AF and varies from one individual to another, and the sensitivity to medications used for rate control therapy differs from patient to patient. Some patients are drug-resistant despite using multiple medications. The determinant of the HR during AF has not yet been clarified. It has been reported that the main determinant of the HR during AF is the conduction characteristics of the atrioventricular (AV) node and autonomic tone^7,8^, but the individual variability of the HR during AF is still not completely elucidated. Genetic differences might explain some of the individual variability in the HR during AF, but there have been a few negative reports about the association of the genetic differences with the HR during AF^9,10^. However, these studies were investigated under a drug administration with a small sample size.

Previous Genome-Wide Association Studies (GWAS) identified 21 single nucleotide polymorphisms (SNPs) associated with the HR during SR^11^, and some of those HR-associated SNPs have been reported to be associated with cardiac conduction. We hypothesized that the HR-associated SNPs reported in the GWAS were also associated with the HR during AF.

## Results

### Relationship between the 21 h-associated SNPs reported in the GWAS and total HR during AF in screening set

**Figure 1:**
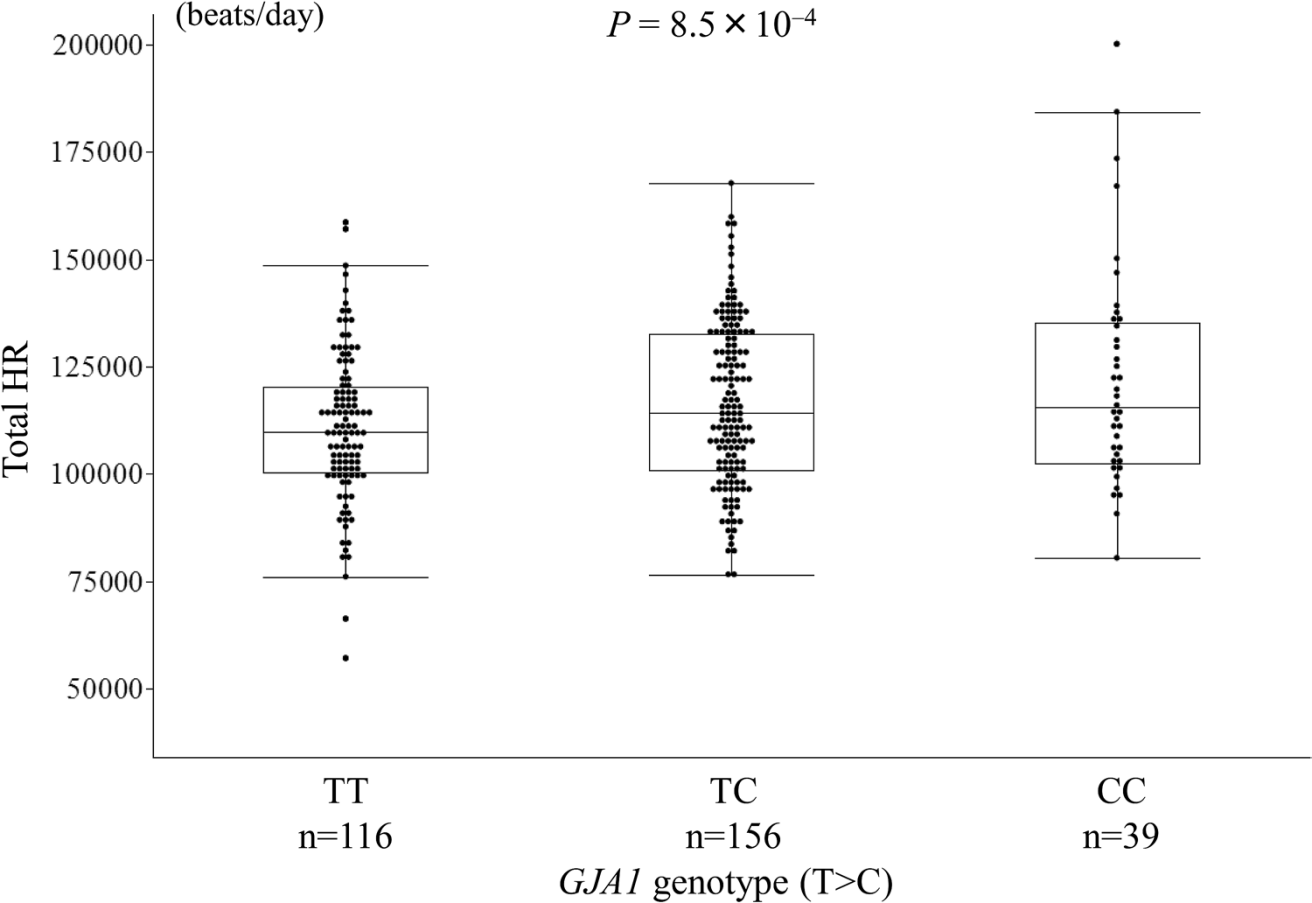
Relationship between the *GJA1* single nucleotide polymorphism (SNP) rs1015451 genotypes and total heart rate (HR) in the patients with persistent atrial fibrillation (AF) in the screening set. The *GJA1* SNP rs1015451 minor allele (C) was associated with a high total HR in the patients with persistent AF in the screening set (TT 110,643 ± 17,542 beats/day, TC 116,350 ± 19,060 beats/day, CC 122,163 ± 25,684 beats/day, *P* = 8.5 × 10^−4^; TT vs CC: *P* = 2.5 × 10^−3^, TT vs TC: *P* = 1.5 × 10^−2^, TC vs CC: *P* = 0.12).

Table 1 shows the relationship between the 21 h-related SNPs reported by the GWAS and the total HR during the 24-h Holter monitoring. The *GJA1* SNP rs1015451 (T>C) genotypes were significantly associated with the total HR after a Bonferroni correction. The total HR during AF was higher in the persistent AF patients with the *GJA1* SNP rs1015451 minor allele than in those without it in the screening set (TT 110,643 ± 17,542 beats/day, TC 116,350 ± 19,060 beats/day, CC 122,163 ± 25,684 beats/day, *P* = 8.5 × 10^−4^; TT vs CC: *P* = 2.5 × 10^−3^, TT vs TC: *P* = 1.5 × 10^−2^, TC vs CC: *P* = 0.12, Fig. 1). The other SNPs were not significantly associated with the total HR. When the relationship between the HR and SNP was examined using the age, gender, and BMI as covariates, no significant SNP other than *GJA1* was observed.

**Table 1:** Relationship between the HR associated SNPs and total HR in the screening set.

Chr	Nearest gene	HR SNP	Alleles	r^2^	P*	Adjusted# P*
14	*MYH6*	rs365990	A>G	5.1 × 10^–6^	0.969	0.767
6	*GJA1*	rs1015451	T>C	0.037	8.5 × 10^–4^	1.3 × 10^–3^
7	*ACHE*	rs13245899	A>G	4.8 × 10^–4^	0.706	0.743
1	*CD46*	rs11118555	T>A	0.002	0.477	0.494
11	*FADS1*	rs174549	G>A	0.003	0.375	0.235
6	*SLC35F1*	rs11153730	T>C	5.3 × 10^–5^	0.900	0.983
12	*LINC00477*	rs17287293	A>G	2.3 × 10^–4^	0.792	0.968
20	*KIAA1755*	rs6127471	C>T	0.002	0.444	0.589
2	*CCDC141*	rs17362588	G>A			
12	*SYT10*	rs7980799	C>A	5.3 × 10^–4^	0.689	0.668
15	*HCN4*	rs4489968	T>G	0.003	0.379	0.328
3	*GNB4*	rs7612445	G>T	1.6 × 10^–4^	0.828	0.805
14	*FLRT2*	rs17796783	T>C	9.4 × 10^–6^	0.958	0.916
7	*CHRM2*	rs2350782	T>C	0.006	0.171	0.098
5	*NKX2-5*	rs6882776	G>A	0.011	0.074	0.029
7	*GNG11*	rs180242	A>T	0.001	0.633	0.547
2	*B3GNT7*	rs13030174	A>C	0.001	0.609	0.948
3	*FNDC3B*	rs9647379	G>C	0.007	0.161	0.213
12	*RFX4*	rs2067615	T>A	0.006	0.166	0.121
12	*CPNE8*	rs826838	T>C	3.8 × 10^–4^	0.738	0.820
2	*TFPI*	rs4140885	G>A	3.3 × 10^–5^	0.922	0.883

*AF* atrial fibrillation, *HR* heart rate, *PAF* paroxysmal atrial fibrillation.

*Uncorrected P value in additive model.

^#^Adjusted by age, gender, and BMI.

R^2^ coefficient of determination.

### Relationship between the clinical characteristics, echocardiographic parameters, and *GJA1* SNP rs1015451 genotypes

The age, gender, body mass index, and duration of AF were similar among the 3 *GJA1* SNP rs1015451 genotypes. The rates of diabetes, hypertension, strokes, structural heart disease, and heart failure were also similar among the 3 *GJA1* SNP genotypes. There were no differences in the left atrial (LA) diameter, LA volume, or left ventricular ejection fraction (LVEF) between the 3 *GJA1* SNP genotypes (Table 2).

**Table 2:** Characteristics and echocardiographic parameters of the patients with AF and the *GJA1* SNP genotypes.

	All	*GJA1* Genotypes (T > C)			*P*
		TT	TC	CC	
		(n = 116)	(n = 156)	(n = 39)	
Age (years)	62.5 ± 9.6	62.8 ± 10.4	62.1 ± 9.0	63.2 ± 8.9	0.99
Male (%)	78.1	79.3	78.2	74.4	0.56
BMI (kg/m^2^)	24.4 ± 3.2	24.2 ± 3.2	24.5 ± 3.3	24.4 ± 3.2	0.53
AF duration (day)	517 ± 895	578 ± 995	488 ± 848	449 ± 727	0.36
Hypertension (%)	58.2	53.5	60.3	64.1	0.17
Diabetes mellitus (%)	19.3	17.2	18.6	28.2	0.21
Stroke (%)	10.3	12.1	7.7	15.4	0.98
Structural heart disease (%)	6.1	6.9	4.5	10.3	0.80
Heart failure (%)	11.9	13.8	10.9	10.3	0.45
LA volume (ml)	82.4 ± 21.6	82.8 ± 20.5	83.5 ± 21.9	76.1 ± 22.7	0.26
LAD (mm)	41.9 ± 6.0	41.5 ± 6.1	42.2 ± 5.9	41.2 ± 6.0	0.81
LVDd (mm)	47.9 ± 4.8	47.8 ± 4.9	48.3 ± 4.7	46.7 ± 4.3	0.53
LVDs (mm)	33.0 ± 4.7	33.0 ± 5.0	33.0 ± 4.5	32.6 ± 4.6	0.66
IVS (mm)	9.2 ± 1.6	9.1 ± 1.7	9.2 ± 1.4	9.3 ± 1.4	0.52
LVEF (%)	56.2 ± 8.7	55.6 ± 9.2	56.9 ± 7.9	55.4 ± 9.5	0.68

*AF* atrial fibrillation, *BMI* body mass index, *IVS* interventricular septum, *LA* left atrial, *LAD* left atrial diameter, *LVDd* left ventricular end-diastolic diameter, *LVDs* left ventricular end-systolic diameter, *LVEF* left ventricular ejection fraction, *SNP* single nucleotide polymorphism.

### Relationship between the EPS parameters and the *GJA1* SNP rs1015451 genotypes

The relationship between the electrophysiological study (EPS) parameters and *GJA1* SNP rs1015451 genotypes is shown in Table 3. The *GJA1* SNP rs1015451 genotypes were significantly associated with the intra-atrial conduction time. The intra-atrial conduction times from the high right atrium (HRA) to the His bundle electrogram (HBE) and from the HRA to the distal coronary sinus (CS) were shorter in the patients with the *GJA1* SNP rs1015451 minor allele than in those without it (HRA to HBE: TT 39.7 ± 14.0 ms, TC 36.8 ± 15.1 ms, CC 29.6 ± 11.1 ms, *P* = 6.1 × 10^−4^, HRA to distal CS: TT 111.6 ± 23.1 ms, TC 108.6 ± 22.2 ms, CC 98.8 ± 18.6 ms, *P* = 6.4 × 10^−3^). Furthermore, the AF cycle length was significantly shorter in the persistent AF patients with the *GJA1* SNP rs1015451 minor allele than in those without it (TT 155 ± 21 ms, TC 149 ± 18 ms, CC 133 ± 16 ms, *P* = 2.2 × 10^−4^). However, the maximum sinus node recovery time (SNRT), corrected SNRT (CSRT), atrial to the His bundle (AH) interval, His bundle to the first ventricular activation (HV) interval, 1:1 AV nodal conduction, and effective refractory period (ERP) of the AV node were similar among the 3 *GJA1* SNP rs1015451 genotypes.

**Table 3:** EPS parameters and the *GJA1* SNP genotypes.

	*GJA1* Genotypes (T > C)			*P*
	TT	TC	CC	
	(n = 116)	(n = 156)	(n = 39)	
Maximum SNRT (ms)	1559 ± 440	1479 ± 446	1468 ± 315	0.15
CSRT (ms)	621 ± 329	578 ± 349	604 ± 267	0.52
1:1 AV nodal conduction (bpm)	140 ± 34	140 ± 27	145 ± 28	0.42
A ERP (ms)	227 ± 30	226 ± 38	234 ± 33	0.18
AV nodal ERP (ms)	346 ± 74	330 ± 83	341 ± 88	0.35
AF cycle length (ms)	155 ± 21	149 ± 18	133 ± 16	2.2 × 10^–4^
**Conduction time**				
HRA to HBE (ms)	39.7 ± 14.0	36.8 ± 15.1	29.6 ± 11.1	6.1 × 10^–4^
HRA to CS distal (ms)	111.6 ± 23.1	108.6 ± 22.2	98.8 ± 18.6	6.4 × 10^–3^
AH interval (ms)	103.0 ± 29.4	103.3 ± 28.3	108.1 ± 37.1	0.48
HV interval (ms)	43.1 ± 10.4	43.0 ± 10.0	45.2 ± 14.7	0.48

*AERP* atrial effective refractory period, *AF* atrial fibrillation, *AH* atrial-His, *AV* atrioventricular, *CS* coronary sinus, *CSRT* corrected sinus node recovery time, *EPS* electrophysiological study, *HBE* His bundle electrogram, *HRA* high right atrium, *HV* His-ventricular, *SNP* single nucleotide polymorphism, *SNRT* sinus node recovery time.

### Multivariate analysis of the total HR during AF in patients with persistent AF

In the univariate analysis, the *GJA1* SNP rs1015451 genotypes, female gender, lower LVEF, higher 1:1 AV nodal conduction, shorter AF cycle length, and shorter atrial conduction time were significantly associated with the higher total HR during AF. A multivariate analysis revealed that the presence of a *GJA1* SNP rs1015451 minor allele C, female gender, lower LVEF, and higher 1:1 AV nodal conduction were independently associated with higher HR during AF (Table 4).

**Table 4:** Clinical and genetic predictors of the total HR during AF.

	Univariate *P*	Multivariate *P*
Age (years)	0.53	
Gender (Men %)	6.7 × 10^–5^	5.2 × 10^–6^
BMI (kg/m^2^)	0.38	
Hypertension (%)	0.65	
Diabetes mellitus (%)	0.28	
Heart failure (%)	0.10	
AF duration (day)	0.49	
LAD (mm)	0.81	
LVDd (mm)	0.20	
LVEF (%)	9.2 × 10^–6^	1.6 × 10^–4^
CSRT (ms)	0.65	
1:1 AV nodal conduction (bpm)	6.7 × 10^–12^	1.8 × 10^–11^
A ERP (ms)	0.66	
AF cycle length (ms)	4.4 × 10^–2^	
Conduction time from HRA to distal CS (ms)	4.9 × 10^–5^	
*GJA1* SNP rs1015451 additive model	8.5 × 10^–4^	1.7 × 10^–2^
*GJA1* SNP rs1015451 dominant model	3.7 × 10^–3^	
*GJA1* SNP rs1015451 recessive model	1.6 × 10^–2^	

*AERP* atrial effective refractory period, *AV* atrioventricular, *BMI* body mass index, *CS* coronary sinus, *CSRT* corrected sinus node recovery time, *HRA* high right atrium, *LAD* left atrial diameter, *LVDd* left ventricular end-diastolic diameter, *LVEF* left ventricular ejection fraction, *SNP* single nucleotide polymorphism.

### Relationship between the *GJA1* SNP rs1015451 genotypes and total HR during AF in the replication set

**Figure 2:**
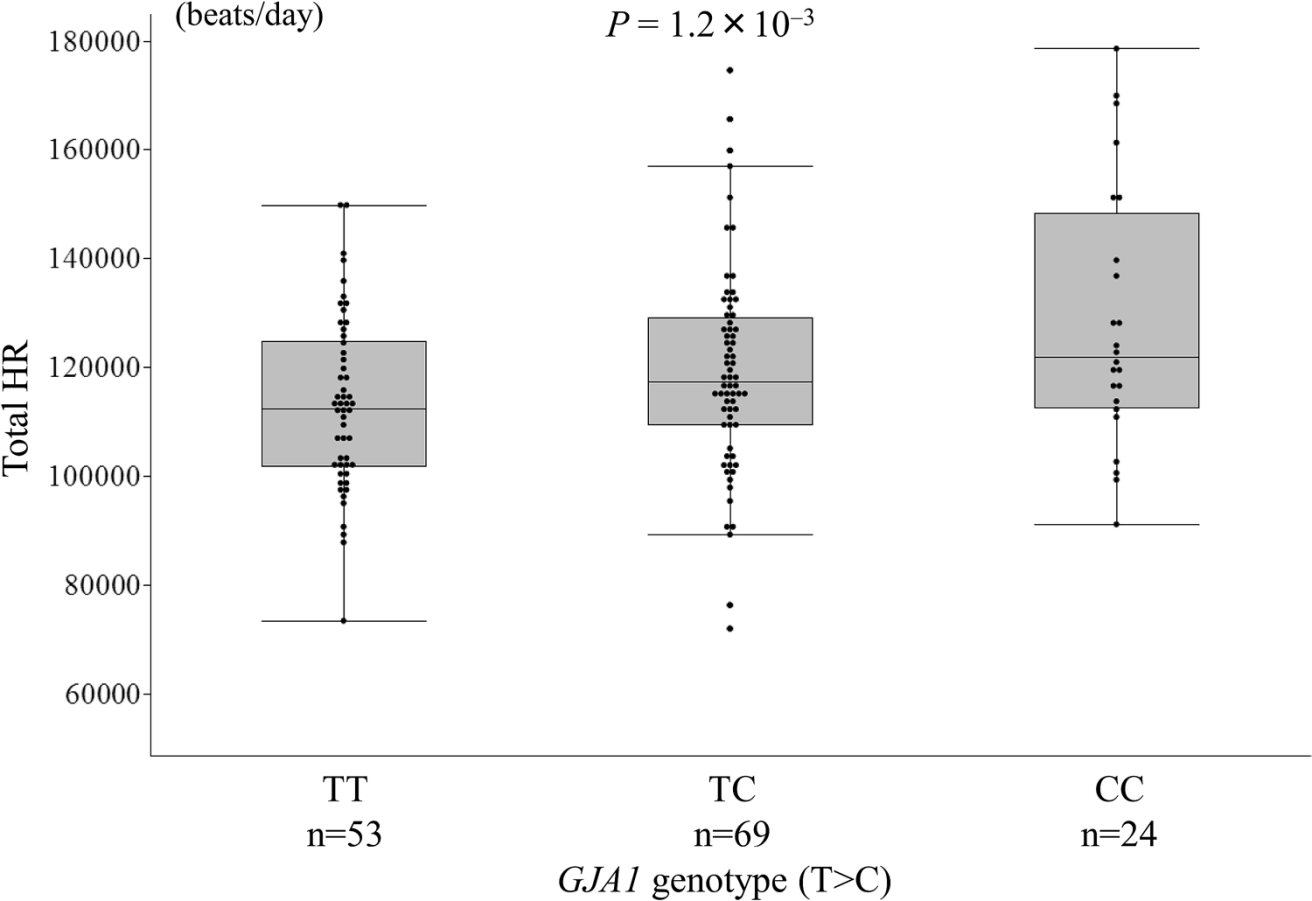
Relationship between the *GJA1* single nucleotide polymorphism (SNP) rs1015451 genotypes and total heart rate (HR) in the patients with persistent atrial fibrillation (AF) in the replication set. The *GJA1* SNP rs1015451 minor allele (C) was associated with a high total HR in the patients with persistent AF in the replication set (TT 113,139 ± 15,761 beats/day, TC 119,014 ± 18,771 beats/day, CC 128,489 ± 23,424 beats/day, *P* = 1.2 × 10^−3^; TT vs CC: *P* = 1.4 × 10^−3^, TT vs TC: *P* = 0.07, TC vs CC: *P* = 0.05).

We confirmed the association between the *GJA1* SNP rs1015451 and total HR during AF in the replication set (TT 113,139 ± 15,761 beats/day, TC 119,014 ± 18,771 beats/day, CC 128,489 ± 23,424 beats/day, *P* = 1.2 × 10^−3^; TT vs CC: *P* = 1.4 × 10^−3^, TT vs TC: *P* = 0.07, TC vs CC: *P* = 0.05, Fig. 2). The total HR during AF was higher in the persistent AF patients with the *GJA1* SNP rs1015451 minor allele than in those without it and also in the replication set. (Fig. 2).

### Relationship between the *GJA1* SNP rs1015451 genotypes and serum MiRNA concentrations

After a quality check, we analyzed 353 miRNAs, in which there were no missing data. To normalize the signals across the different microarrays tested, Norm Finder software was used to assess the stability of the miRNAs. The hsa-miR-149-3p was selected as a normalizer.

We investigated the relationship between the *GJA1* SNP rs1015451 genotypes and plasma concentrations of the 353 miRNAs. Although only two miRNAs, miR-4708-3p (*P* = 4.6 × 10^−2^) and miR-4448 (*P* = 4.6 × 10^−2^), had *P* values of < 0.05, those were not significantly associated with *GJA1* SNP rs1015451 genotypes after a Bonferroni correction. The top 10 miRNAs are shown in the Supplementary Table.

## Discussion

In our study, the *GJA1* SNP rs1015451 (T>C) minor allele was associated with a higher HR, shorter atrial conduction time, and shorter AF cycle length in the patients with persistent AF.

AF is the most common cardiac arrhythmia that causes tachycardia and heart failure^1–3^. The HR during AF differs from person to person, and many factors are involved in the AF HR. The determinants of the AF HR have not been completely identified. Also, the genetic background of individual HR variations during AF has not been examined. The 21 SNPs associated with the HR during sinus rhythm have been reported by a previous GWAS^11^. We investigated whether those SNPs were also associated with the HR during AF in patients with persistent AF. We initially clarified that only the *GJA1* SNP rs1015451 (T>C) minor allele was associated with a higher HR during AF in patients with persistent AF. As a pertinent issue, the *GJA1* SNPs including rs1015451 have recently been reported to be associated with heart failure and a reduced ejection fraction^12^.

Cardiac conduction is mediated by gap junction channels formed by connexin protein subunits. The *GJA1* gene encodes connexin-43 (Cx43), which is one of the main connexin proteins^13,14^. Three types of connexins, Cx40, 43, and 45, are expressed differently depending on the site of the human cardiomyocytes^15,16^. Cx43 is the most expressed connexin in the atrial working myocardium, and it is also abundantly expressed in the working ventricular myocardium. Cx40 is abundantly expressed in the intraventricular conduction systems that control fast conduction, such as in the His bundle and Purkinje fibers. Cx45 is mainly expressed in the sinoatrial node and AV node, which have slow conduction properties. On the other hand, the expression level of Cx43 at the sinus node and AV node is very low^16,17^. In our study, the *GJA1* SNP rs1015451 was associated with the conduction time of the atria, but it was not associated with the AV node conduction or SNRT. Those results were consistent with the localization of Cx43.

The *GJA1* SNP rs1015451 minor allele (C) was also associated with a short AF cycle. Many studies on the AF cycle length have used an F-wave frequency analysis of the surface electrocardiography (ECG), and studies using an AF cycle length measurement from the intracardiac ECG have been few^18,19^. These two methods are reported to be correlated^20,21^, but the intracardiac ECG is more accurate. We analyzed the intracardiac ECG to evaluate the AF cycle length accurately. The HR during AF is influenced by many factors, such as the conduction characteristics of the AV node and autonomic tone. In our study, a shorter AF cycle was also associated with a higher HR during AF. The *GJA1* SNP rs1015451 minor allele was associated with a higher HR during AF through a faster atrial conduction and shorter AF cycles, independent of the AV node conduction.

The SNP rs1015451 is called the *GJA1* gene SNP^11^ because the *GJA1* gene is the closest gene. However, the *GJA1* SNP rs1015451 is located outside of the *GJA1* gene, approximately 370 kB away from the *GJA1* gene. Variants that are in a strong linkage disequilibrium (LD) with the *GJA1* SNP rs1015451 (r^2^ > 0.8), are located in uncharacterized *LOC105377979* and do not spread to the *GJA1* gene (Supplementary Fig. 1). Previous studies have suggested that variations in the intergenic regions might regulate transcription factor binding and chromatin modification^22^. We investigated the expression quantitative trait locus (eQTL) data acquired from 429 human LA appendage samples, 432 human left ventricle samples and 532 human peripheral nerves available from the Genotype-Tissue Expression (GTEx) website (http://gtexportal.org; V7 release) for the cis-eQTL effects of *GJA1* SNP rs1015451. We analyzed the genes located within 1 Mb upstream and downstream to *GJA1* SNP rs1015451, but we found no genes, including *GJA1*, for which the expression was significantly associated with the *GJA1* SNP rs101545. In addition, there were no LD SNPs around the *GJA1* SNP rs101545 associated with the *GJA1* expression (Supplementary Fig. 2-1,2). On the other hand, the expression level of the *GJA1* gene in the peripheral nerves was significantly higher in patients with the *GJA1* SNP rs1015451 minor C allele (Supplementary Fig. 2-3), suggesting that epigenetic regulation could be involved in this process. We investigated the relationship between the *GJA1* SNP rs1015451 genotypes and plasma concentrations of 353 miRNAs, and they were not significantly associated with the *GJA1* SNP rs1015451 genotypes. More functional studies focused on this intergenic region on chromosome 6q22 will be required to understand its potential effect on *GJA1*.

There have been some negative reports about the association of the genetic SNPs with AF rate control. Barret et al. reported that no SNP was associated with acute HR control after the administration of diltiazem^9^. Kolek et al. reported that no SNP was significantly associated with a poor AF HR control^10^. In our study, the *GJA1* gene SNP was associated with the HR during AF in patients with persistent AF in screening and replication sets.

To the best of our knowledge, this is the first report to demonstrate that for the *GJA1* gene, encoding the gap junction Cx43, the SNP is associated with the HR during AF. This finding has a clinical implication in that early detection of high-risk AF patients with AF tachycardias who are prone to develop heart failure, and early intervention in these patients are needed to avoid heart failure.

This study had some study limitations. It was a retrospective study conducted at a single center, and the total number of cases was small, and therefore the statistical power of this study was inadequate. Therefore, we must investigate the association between the HR during AF and these SNPs using a larger sample of cases in future research.

## Conclusion

The *GJA1* gene, encoding the gap junction protein (Cx43), SNP rs1015451 minor allele is associated with a high HR in AF patients. The *GJA1* SNP rs1015451 might be a new genetic marker for AF tachycardia.

## Methods

### Participants

This was a single-center retrospective study. We enrolled 311 patients (243 men and 68 women, mean age 63 ± 10 years) with persistent AF who underwent radiofrequency catheter ablation (RFCA) at the Hiroshima University Hospital from November 2009 to March 2016 for screening. We also enrolled 146 consecutive Japanese persistent AF patients (114 men, 32 women; mean age, 61 ± 10 years) who underwent RFCA at the Hiroshima University Hospital from April 2016 to July 2018 for replication. Persistent AF was defined as AF that lasted longer than 7 days. All procedures involving the human genome use were approved by the Institutional Ethics Committee of the Graduate School of Biomedical Science at the Hiroshima University. Written informed consent was obtained from all participants prior to participation in the study. All methods were performed in accordance with the relevant guidelines and regulations.

### Genotyping

We obtained peripheral blood from all participants and extracted the genomic DNA from leukocytes using the QIAamp DNA Blood Mini Kit (QIAGEN, Hilden, Germany) according to the standard protocol. For all subjects, we genotyped the 21 h-associated SNPs reported in the GWAS (*MYH6*, *GJA1*, *ACHE*, *CD46*, *FADS1*, *SLC35F1*, *LINC00477*, *KIAA1755*, *CCDC141*, *SYT10*, *HCN4*, *GNB4*, *FLRT2*, *CHRM2*, *NKX2-5*, *GNG11*, *B3GNT7*, *FNDC3B*, *RFX4*, *CPNE8*, *TFPI*) using a TaqMan assay.

### Echocardiography

Transthoracic echocardiography was performed at our institution using a commercially-available system (Vivid E9, GE Healthcare, Milwaukee, WI, USA; or iE33, Philips Medical Systems, Andover, MA, USA) before the RFCA. Experienced echocardiographers who were blinded to the genotyping results conducted all echocardiographic examinations and analyzed the echocardiographic parameters.

### Twenty-four-hour Holter monitoring

Twenty-four-hour Holter monitoring was performed one day before the RFCA. Antiarrhythmic drugs, including β-blockers and calcium channel blockers, were stopped at least five half-lives before performing the 24-h Holter monitoring. Only amiodarone was routinely discontinued at least 2 weeks prior to performing the 24-h Holter monitoring. We confirmed that all the subject were AF all the time. The total HR during the AF was recorded in all of the subjects.

### Electrophysiological study and RFCA

We also confirmed that all the patients were AF at the beginning of their AF ablation.

The AF cycle length was recorded on the LA posterior wall and four pulmonary veins. An average cycle length of 5 s before the pulmonary vein isolation (PVI) was calculated. We defined the shortest cycle length of them all as the AF cycle length.

A continuous PVI was performed in all subjects using an open-irrigation 3.5-mm tip deflectable catheter (THERMOCOOL SMARTOUCH; Biosense Webster) under the guidance of a 3-dimensional electro-anatomical mapping system (CARTO3, Biosense Webster) with computed tomography integration (CARTOMERGE, Biosense Webster) to achieve electrical isolation of the left- and right-sided pulmonary veins. We confirmed the PVI entrance and exit block and then rechecked these under the infusion of isoproterenol plus adenosine triphosphate^23^.

After the PVI, an EPS was performed during stable SR. Four 5-French-gauge catheters were used: one catheter was a 10-polar electrode catheter positioned in the CS, and three catheters were quadripolar electrode catheters positioned in the HRA, His bundle, and right ventricle, all with a 5-mm interelectrode distance. The AH and HV intervals were measured during the baseline intracardiac ECG. The SNRT was measured as the recovery interval after a 30-s stimulation from the HRA. The CSRT was defined as the recovery interval in excess of the sinus cycle (i.e., CSRT = maximum SNRT − sinus cycle length)^23^. The ERP was measured using extrastimulus pacing with varied coupling intervals at a basic cycle length of 600 ms. The ERP was defined as the longest coupling interval that failed to propagate through that tissue. The maximal rate of 1:1 AV nodal conduction was determined using incremental atrial pacing.

### miRNA

We investigate the relationships between the *GJA1* SNP rs1015451 and serum concentrations of 2555 miRNAs. The total RNA was extracted from individual serum samples using the 3D-Gene RNA Extraction Reagent from a liquid sample kit (Toray Industries, Inc., Kanagawa, Japan). A total of 2555 miRNA sequences were detected using the 3D-Gene miRNA Labeling kit and 3D-Gene Human miRNA Oligo Chip (Toray Industries, Inc). We analyzed the relationships between the *GJA1* SNP rs1015451 genotype and serum concentrations of the miRNAs in the AF patients^24^.

### Statistical analysis

Continuous are presented as the mean ± standard deviation. The deviation from the Hardy–Weinberg equilibrium was tested among the cases and controls using an ordinary χ^2^ test. An additive mode of inheritance was assumed where the SNPs were coded as 0, 1, and 2 in the linear regression model. The association between the *GJA1* SNP rs1015451 genotype and serum concentrations of the miRNAs was also analyzed by the linear regression model where the SNPs were coded as 0, 1, and 2. A multivariate analysis was performed by means of multiple regression analysis. The Bonferroni-corrected *P*-value threshold was *P* < 0.002 (0.05/21 SNPs) for relationships between the 21 h-associated SNPs and HR in the AF patients. Values of *P* < 0.05 were considered significant for the other analyses. The statistical analyses were conducted using the R3.3.1 and the JMP statistical package (version 13, SAS Institute, Cary, NC).

## Supplementary Information

Supplementary Information

## Data Availability

The data in this study are available from the corresponding author upon reasonable request.
