# Supplementary material for: Minor allele of GJA1 gene polymorphism is associated with higher heart rate during atrial fibrillation
Source: Sci Rep. 2021 Jan 28;11:2549. doi: 10.1038/s41598-021-82117-3 (PMC7844413; doi:10.1038/s41598-021-82117-3)
Supplement: Supplementary file 1 — Supplementary Information [file 41598_2021_82117_MOESM1_ESM.pdf]

Minor Allele of *GJA1* Gene Polymorphism is associated with Higher Heart Rate during Atrial Fibrillation

Sho Okamura[1)], Yuko Onohara[1)], Hidenori Ochi[2),3)], Takehito Tokuyama[1)]
Naoya Hironobe[1)], Yousaku Okubo[1)], Yoshihiro Ikeuchi[1)], Shunsuke Miyauchi[1)]
Kazuaki Chayama[3)], Yasuki Kihara[1)], Yukiko Nakano[1)]

1) Department of Cardiovascular Medicine, Graduate School of Biomedical and Health Sciences, Hiroshima University, Japan
2) Department of Health Management, Hiroshima Red Cross Hospital & Atomic-bomb Survivors Hospital, Japan
3) Department of Gastroenterology and Metabolism, Biomedical Sciences, Graduate School of Biomedical and Health Sciences, Hiroshima University, Japan

Supplementary Figure 1. Linkage disequilibrium of the *GJA1* SNP rs1015451.

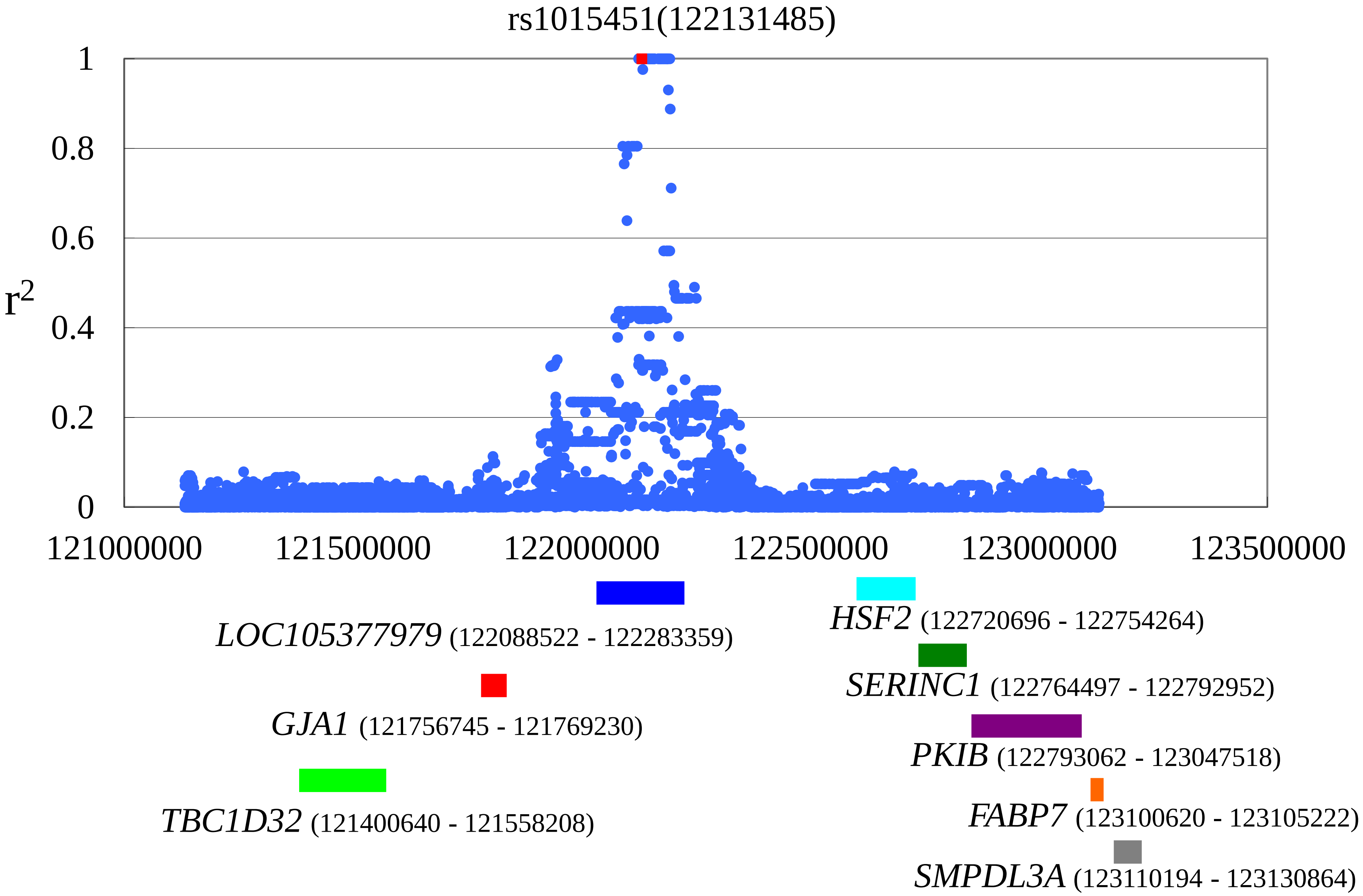

Supplementary Figure 2-1.
Relationship between the *GJA1* expression in the left ventricle and linkage disequilibrium SNPs around *GJA1* SNP rs101545.

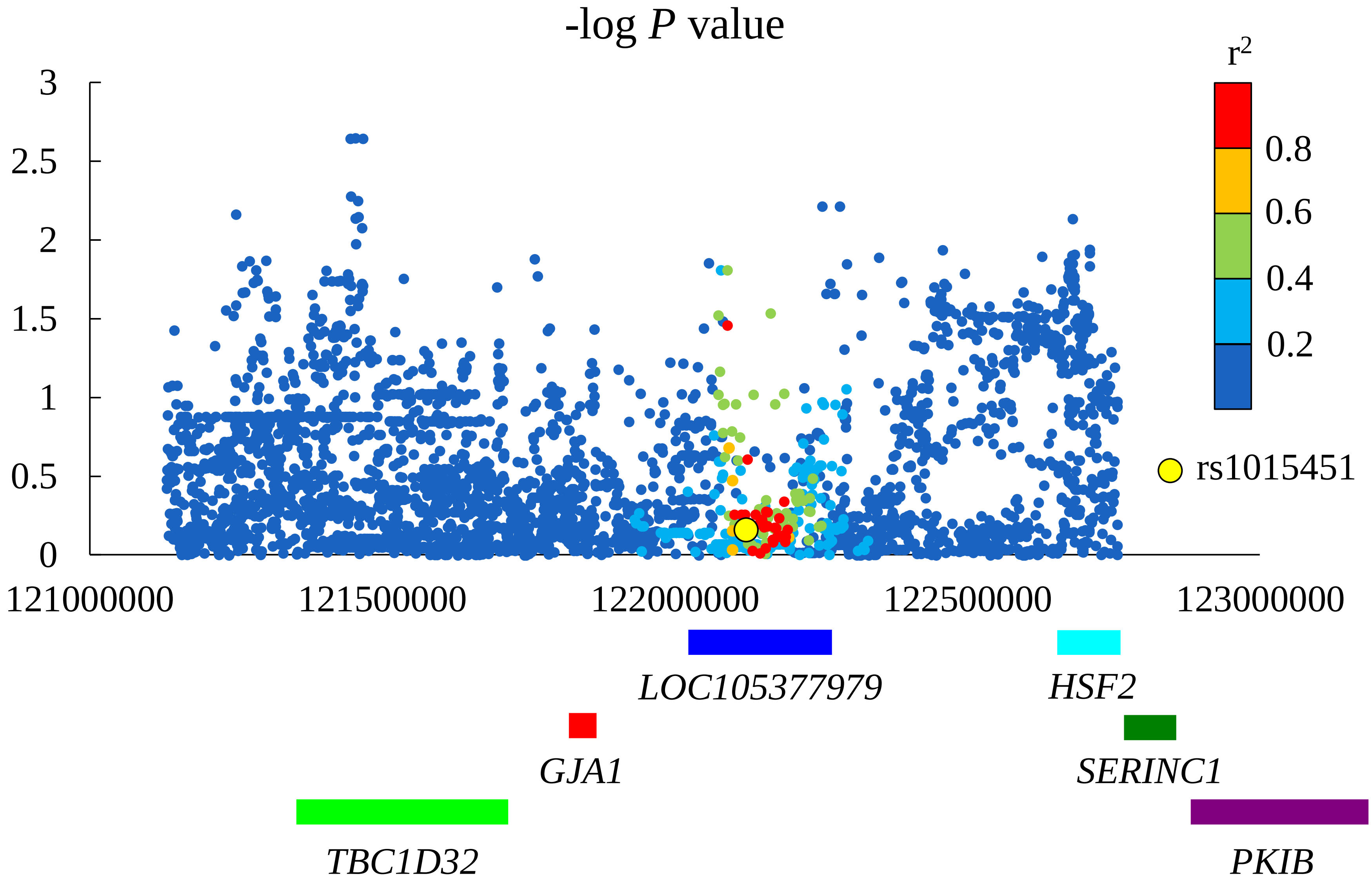

Supplementary Figure 2-2.
Relationship between the *GJA1* expression in the atrial appendage and linkage disequilibrium SNPs around *GJA1* SNP rs101545.

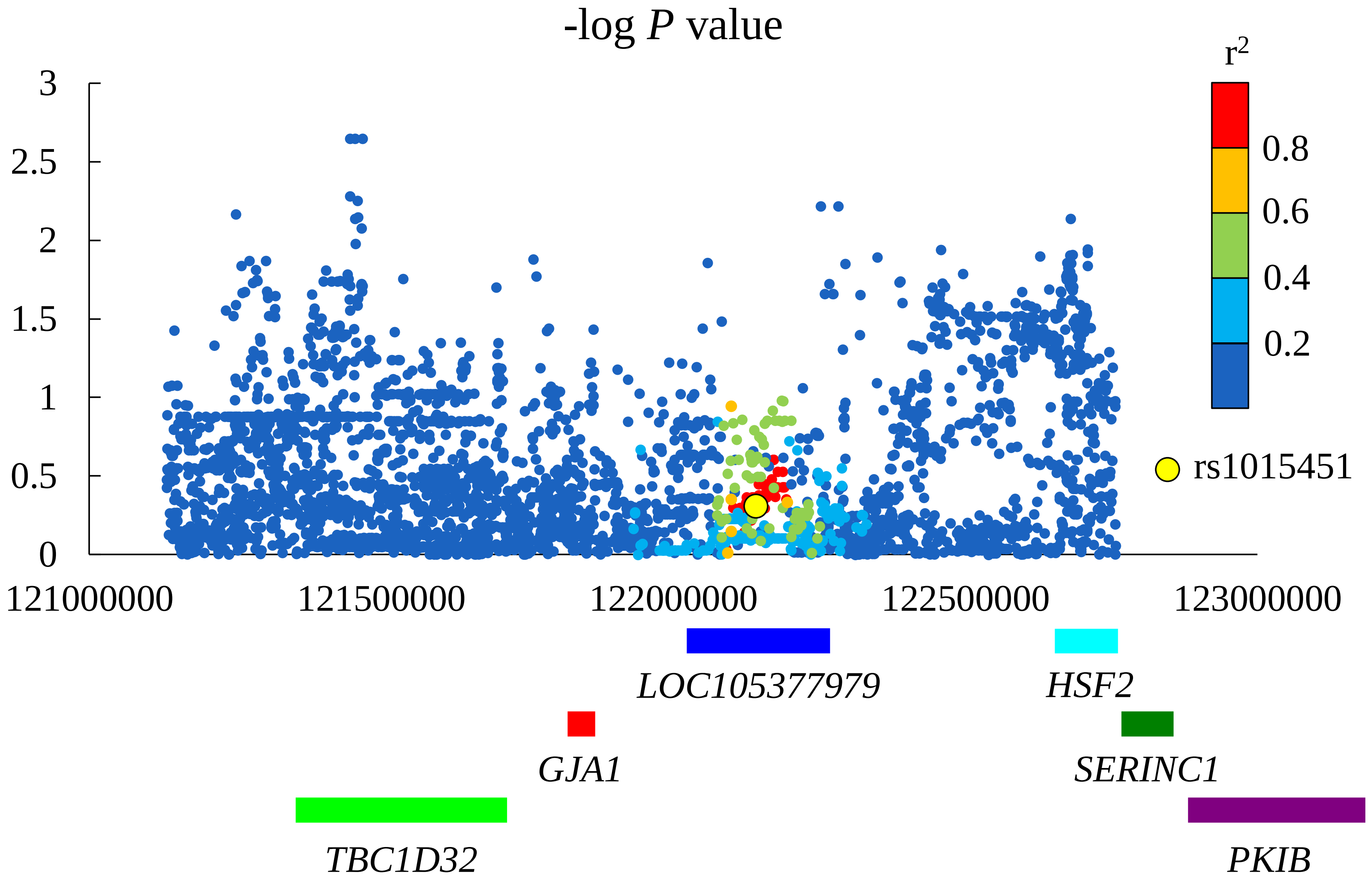

Supplementary Figure 2-3.
Relationship between the *GJA1* expression in the peripheral nerves and linkage disequilibrium SNPs around *GJA1* SNP rs101545.

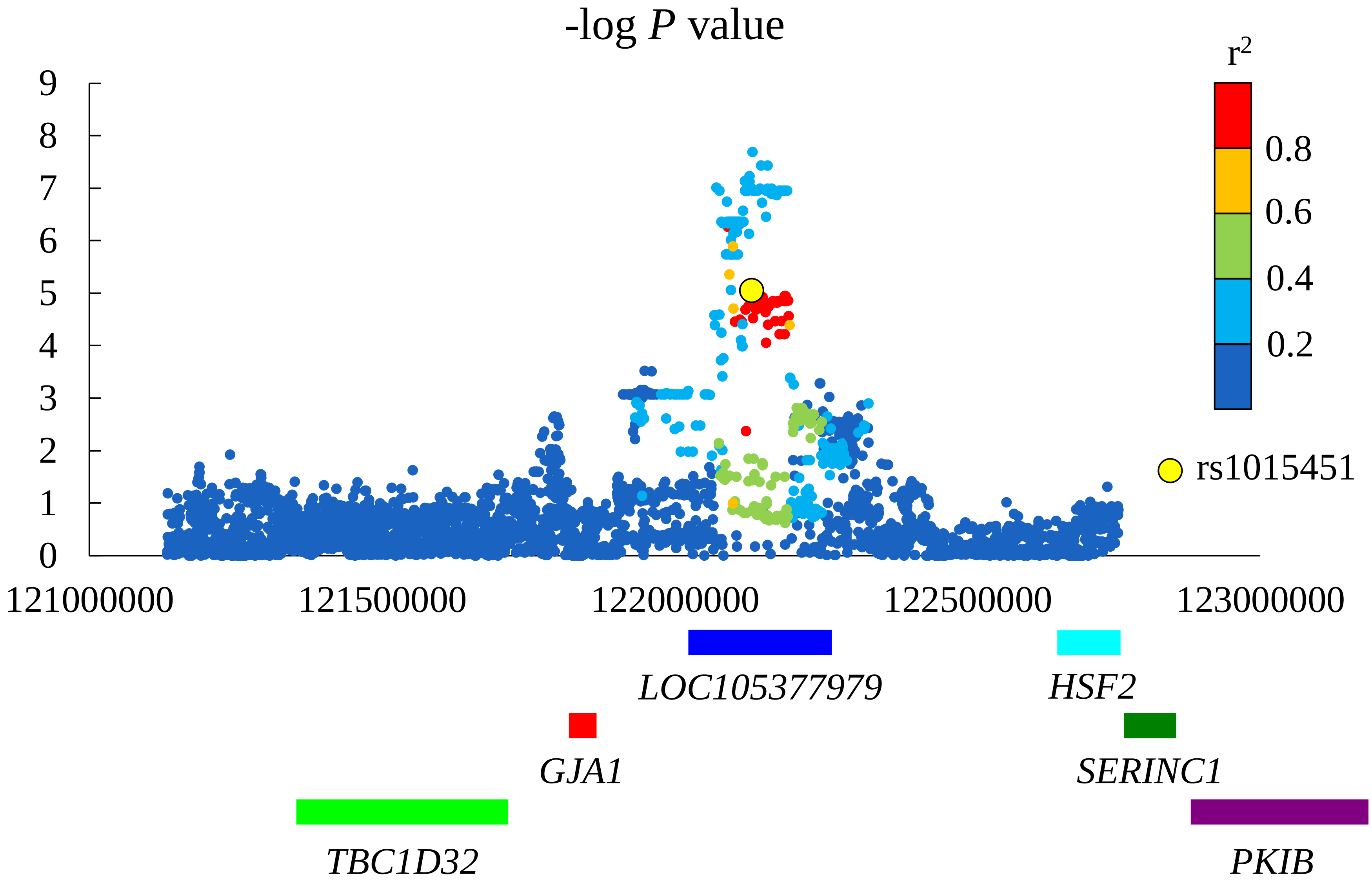

Supplementary Table.
The association between *GJA1* SNP rs1015451 genotype and microRNA serum concentration (log2).

	GJA-1 Genotypes(T > C)			
micro RNA	TT (n = 182)	TC (n = 253)	CC (n = 95)	*P*
miR-4708-3p	190 ± 147	209 ± 165	232 ± 182	0.046
miR-4448	259 ± 215	272 ± 224	324 ± 305	0.046
miR-2467-3p	271 ± 217	297 ± 243	315 ± 245	0.134
miR-4648	517 ± 1933	335 ± 629	316 ± 416	0.143
miR-128-1-5p	1178 ± 778	1220 ± 787	1320 ± 840	0.176
miR-345-3p	307 ± 238	345 ± 321	345 ± 303	0.189
miR-3177-5p	119 ± 58	127 ± 62	128 ± 59	0.192
miR-4258	1547 ± 670	1617 ± 705	1654 ± 722	0.207
miR-4718	131 ± 144	143 ± 174	158 ± 184	0.219
miR-6840-3p	1366 ± 864	1461 ± 899	1486 ± 884	0.225
